# Epileptic pilocarpine‐treated rats exhibit aberrant hippocampal EPSP‐spike potentiation but retain long‐term potentiation

**DOI:** 10.14814/phy2.13490

**Published:** 2017-11-15

**Authors:** Ezekiel Carpenter‐Hyland, Edyta K. Bichler, Mathew Smith, Robert S. Sloviter, Morris Benveniste

**Affiliations:** ^1^ Neuroscience Institute Morehouse School of Medicine Atlanta Georgia; ^2^ Present address: Veterans Affairs Medical Center Atlanta Georgia; ^3^ Present address: Department of Anesthesiology Emory University School of Medicine Atlanta Georgia

**Keywords:** Epilepsy, E‐S plasticity, long‐term potentiation, spike‐timing‐dependent plasticity

## Abstract

Hippocampal neuron plasticity is strongly associated with learning, memory, and cognition. In addition to modification of synaptic function and connectivity, the capacity of hippocampal neurons to undergo plasticity involves the ability to change nonsynaptic excitability. This includes altering the probability that EPSPs will generate action potentials (E‐S plasticity). Epilepsy is a prevalent neurological disorder commonly associated with neuronal hyperexcitability and cognitive dysfunction. We examined E‐S plasticity in chronically epileptic Sprague–Dawley rats 3–10 weeks after pilocarpine‐induced *status epilepticus*. CA1 neurons in hippocampal slices were assayed by whole‐cell current clamp to measure EPSPs evoked by Schaffer collateral stimulation. Using a weak spike‐timing‐dependent protocol to induce plasticity, we found robust E‐S potentiation in conjunction with weak long‐term potentiation (LTP) in saline‐treated rats. In pilocarpine‐treated rats, a similar degree of LTP was found, but E‐S potentiation was reduced. Additionally, the degree of E‐S potentiation was not correlated with the degree of LTP for either group, suggesting that they independently contribute to neuronal plasticity. E‐S potentiation also differed from LTP in that E‐S plasticity could be induced solely from action potentials generated by postsynaptic current injection. The calcium chelating agent BAPTA in the intracellular solution blocked LTP and E‐S potentiation, revealing the calcium dependence of both processes. These findings suggest that LTP and E‐S potentiation have overlapping but nonidentical mechanisms of inducing neuronal plasticity that may independently contribute to cognitive disruptions observed in the chronic epileptic state.

## Introduction

Epilepsy‐associated cognitive dysfunction can cause a significant reduction in the quality of life. Although pathological hyperexcitability leads to seizure generation and ultimately may cause cognitive dysfunction (Helmstaedter et al. 2003; Motamedi and Meador 2003; Elger et al. 2004; Hermann et al. 2006, 2010), their underlying biological mechanisms may differ. Growing evidence suggests that the development of epilepsy involves cellular mechanisms with similarities to those invoked in neuroplasticity (Beck et al. 2000; Bernard et al. 2004; Klatte et al. 2013). Our overall aim is to better understand potential relationships between neuroplastic changes that take place in the chronically epileptic state and their associated cognitive dysfunctions.

Long‐term potentiation (LTP) is a widely studied physiological paradigm for synaptic plasticity, and a model of memory formation (Malenka and Bear 2004; Kessels and Malinow 2009; Bliss and Collingridge 2013; Bliss et al. 2014). Investigations of human temporal lobe epilepsy tissue (Beck et al. 2000) and electrical, chemical, and hypoxia‐induced animal models suggest that seizures can lead to a short‐term increase in synaptic strength at hippocampal Schaffer collateral‐CA1 pyramidal cell synapses. However, days to weeks after *status epilepticus*, a reduction or elimination of LTP can be observed (Reid and Stewart 1997; Zhang et al. 2010; Zhou et al. 2011; Suarez et al. 2012). Despite these findings, the molecular pathways that directly produce cognitive dysfunction during chronic epilepsy remain unidentified.

An additional way neurons exhibit plasticity is through modification of intrinsic excitability. This extrasynaptic form of plasticity can be generated through persistent changes in dendritic integration and/or action potential (AP) firing via regulation of ionic conductances in nonsynaptic compartments (reviewed in Daoudal and Debanne 2003; Zhang and Linden 2003). One functional measure of a change in extrasynaptic excitability is alteration of the relationship between excitatory postsynaptic potential (EPSP) generation and generation of APs (spikes). This relationship is termed “E‐S plasticity.” Field potential recordings have indicated that the CA1 region of the hippocampus yields an increase in population spike amplitude for similar sized field EPSPs after the Schaffer collateral pathway is tetanized (Andersen et al. 1980; Abraham et al. 1987; Chavez‐Noriega et al. 1990). This form of E‐S potentiation has been found to be NMDA receptor‐dependent (Jester et al. 1995; Daoudal et al. 2002; Wang et al. 2003; Xu et al. 2005; Campanac and Debanne 2008; Fink and O'Dell 2009). Although altered intrinsic excitability has been reported for the CA1 region when seizure models are employed (Peters et al. 2005; Zhang et al. 2006; Jung et al. 2007; Lopez de Armentia et al. 2007; Shah et al. 2008; Shin et al. 2008; Su et al. 2008), the ease of induction of E‐S plasticity in epilepsy models has not been studied.

Here we determined whether E‐S plasticity is dysfunctional in chronically epileptic animals. Using the pilocarpine model of temporal lobe epilepsy in young adult rats, we stimulated the Schaffer collateral pathway using plasticity‐inducing protocols to examine synaptic and extrasynaptic excitability changes in CA1 pyramidal neurons. Although LTP was not significantly different in neurons from chronically epileptic rats and saline‐treated controls, E‐S potentiation was abnormal in cells from the chronically epileptic animals. These findings suggest that E‐S potentiation is independent of LTP, and is altered in the chronic epileptic state. E‐S plasticity may therefore be a contributor to cognitive comorbidities in epilepsy.

## Methods

### Ethical approval

All procedures were performed under the supervision and approval of the Morehouse School of Medicine Institutional Animal Care and Use Committee. Sprague–Dawley male rats (Envigo, Indianapolis, IN) were housed, 2–4 animals per cage in 12:12 h light–dark cycle and fed ad libitum prior to pilocarpine treatment.

### Pilocarpine treatment

The age of rats chosen for pilocarpine treatment (P40 to P45) served as an optimal time window when pilocarpine injection would produce reliable *status epilepticus*, with rats still being young enough to allow for successful whole‐cell recording after a 3‐ to 10‐week waiting period to ensure a chronic epileptic state (mean time after *status epilepticus*: 35.8 ± 1.2 days, *n* = 13 cells from eight animals). We did not wait longer periods due to the increased difficulty of achieving hour‐long, whole‐cell recordings from older epileptic rats. Rats were first injected with 2 mg/kg (s.c.) each of methylscopolamine and terbutaline prepared in sterile 0.9% saline. Thirty minutes later, *status epilepticus* was induced by injection of pilocarpine (385 mg/kg, i.p.) dissolved in sterile 0.9% saline. Greater than 90% of rats entered behavioral *status epilepticus*, determined as Stage 5 by the Racine scale (Racine 1972). These seizures are characterized by multiple behaviors including rearing and falling, head nodding, forelimb clonus, and loss of posture. Upon reaching Stage 5, rats continuously maintained *status epilepticus* for 90 min, after which 20 mg/kg i.p. pentobarbital was injected to stop behavioral expression of seizures. The few pilocarpine‐treated rats that did not reach Stage 5 or could not sustain 90 min of *status epilepticus* were excluded. Vehicle controls were injected with saline and treated with pentobarbital after 90 min. Animals were monitored for several hours, and hydrated by subcutaneous injection of 0.9% sterile saline. Rats were housed individually after pilocarpine treatment, and received moistened food pellets in the cage for the first 48 h. This procedure produces a form of chronic epilepsy with spontaneous behavioral seizures developing reliably within days after the end of *status epilepticus* (Klitgaard et al. 2002; Harvey and Sloviter 2005). Thus, the 3‐ to 10‐week time window chosen for whole‐cell experiments captured animals in the chronic epileptic state.

### Fluoro‐Jade B and Nissl Histology

Under urethane anesthesia, rats were perfused through the aorta with saline for 2 min, followed by 4% paraformaldehyde in 0.1 mol/L phosphate buffer, pH 7.4, for 10 min. The intact rats were stored overnight at 4°C. This was followed by brain extraction and 50‐*μ*m‐thick horizontal sections were cut in 0.1 mol/L Tris buffer, pH 7.6, using a vibratome. Serial sections were mounted on Superfrost Plus slides for subsequent Nissl (1% cresyl violet) and Fluoro‐Jade B staining (Schmued and Hopkins 2000). Subsequently, specimens were dehydrated with ethanol, the ethanol replaced by xylene, and coverslipped with Permount. All histological and staining procedures have been described previously (Bumanglag and Sloviter 2008). Brightfield and fluorescence specimens were imaged digitally using a Nikon E800M microscope with a Nikon DS‐Fi2 digital camera and Nikon NIS‐Elements microscope imaging software. Images were prepared for display using GIMP 2.8.18 software (GNU Image Manipulation Program, open‐source from the GIMP team at www.gimp.org).

### In Vitro slice electrophysiology

P60–P115 rats were placed in a sealed chamber and anesthetized using isoflurane until areflexic. Transcardial perfusion was then performed under isoflurane anesthesia using an ice‐cold perfusion solution containing 200 mmol/L sucrose, 2.5 mmol/L KCl, 1.2 mmol/L NaH_2_PO_4_, 25 mmol/L NaHCO_3_, 20 mmol/L D‐glucose, 0.5 mmol/L CaCl_2_, 7.0 mmol/L MgCl_2_, 2.4 mmol/L Na‐Pyruvate, 1.3 mmol/L L‐ascorbic acid and oxygenated by bubbling with 95% O_2_/5% CO_2_. Anesthetized rats were decapitated, the brains rapidly removed and 350 *μ*m horizontal hippocampal slices made in ice‐cold oxygenated perfusion solution with a VT1000S vibratome (Leica Biosystems, Buffalo Grove, IL). Slices were transferred to a preincubation chamber containing oxygenated ACSF (125 mmol/L NaCl, 2.8 mmol/L KCl, 1.0 mmol/L NaH_2_PO_4_, 26 mmol/L NaHCO_3_, 10 mmol/L glucose, 2.0 mmol/L CaCl_2_, 1.5 mmol/L MgSO_4_) and warmed to 32°C for 20 min before being returned to room temperature (Hoffman and Johnston 1998).

Prior to electrophysiological recording, the CA1 region was isolated by a diagonal cut between CA1 and CA3 subfields through the CA3 *stratum radiatum*. Whole‐cell current clamp recordings were performed utilizing an EPC10 amplifier (HEKA Instruments, Holliston, MA) on CA1 pyramidal neurons at 32°C. During recordings, slices were bathed in oxygenated ACSF containing 30**** *μ*mol/L bicuculline and 1 *μ*mol/L CGP‐55845 to block GABA_A_ and GABA_B_ receptor activity, respectively. Thick wall patch clamp electrodes (8–12 MΩ) were filled with intracellular solution containing 117.5 mmol/L K‐gluconate, 20 mmol/L KCl, 10 mmol/L HEPES, 0.5 mmol/L EGTA, 7 mmol/L Na‐creatine phosphate, 2.0 mmol/L MgATP, 0.3 mmol/L Na_2_GTP, pH 7.3 and 280 mOsm. In some experiments, a BAPTA intracellular solution was used containing 75 mmol/L K‐gluconate, 20 mmol/L KCl, 10 mmol/L HEPES, 30 mmol/L K_4_BAPTA, 4 mmol/L NaCl, 2 mmol/L MgATP, pH 7.3 and 280 mOsm.

**Figure 1:**
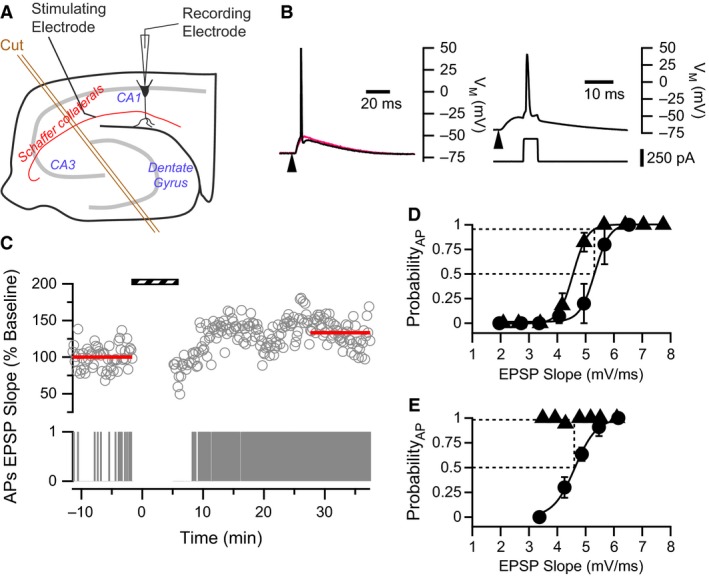
Measuring P_50_ Firing to gauge E‐S potentiation in CA1 neurons. (A) Schematic diagram showing the configuration for hippocampal slice experiments, noting Schaffer collateral stimulation and CA1 whole‐cell recording sites. An incision made to isolate the CA1 circuit is also depicted (orange double lines). (B) Examples of evoked EPSPs from representative CA1 pyramidal cells. *Left*: Test pulses consisted of stimulation to the Schaffer collaterals (arrowhead) that evoked EPSPs. Two such EPSPs with similar initial slopes are shown. The same stimulus intensity was used to evoke both EPSPs; however, 30–40% of the time, EPSPs were suprathreshold and evoked an AP (black trace), whereas other times EPSPs were subthreshold (red trace). *Right*: Pairing protocol. Upper voltage trace shows an evoked EPSP followed by an AP which was evoked by a current pulse (lower trace) delivered to the postsynaptic cell soma with an 8 ms delay. Arrowhead indicates stimulation of the Schaffer collaterals to evoke the EPSP. (C) Example showing that WPP yielded a modest increase in EPSP slope and a strong increase in AP firing. Upper trace represents EPSP slopes normalized to the baseline. Lower panel is a binary raster plot of AP Firing. In the AP raster plot, 1 represents the presence of an AP evoked by a suprathreshold EPSP, whereas 0 represents the absence of an AP. Stripped bar indicates when WPP was applied. (D & E) P_50_ Firing values reflect changes in E‐S coupling. Two examples of E‐S relationships before (filled circles) and after (filled triangles) WPP. In both examples, data were collected utilizing a range of stimulus intensities applied to the Schaffer collaterals. (D) An example where variability in sub‐ and suprathreshold EPSPs generates E‐S curves with a sigmoidal relationship. Solid lines represent fits to Equation 1. Fitted parameters before WPP: ES_50_ = 5.3 mV/ms, *n* = 0.27; after plasticity induction: ES_50_ = 4.6 mV/ms, *n* = 0.25. The dotted lines illustrate how P_50_ Firing values are calculated (*see* Methods). P_50_ Firing in this neuron is 0.95. (E) In some CA1 neurons from saline‐treated rats, complete E‐S curves could not be constructed after WPP because of the dramatic increase in (E‐S) coupling. Yet, P_50_ Firing values still reflect shifts in E‐S coupling after the induction protocol. Fitted parameters (Equation 1) before plasticity induction: ES_50_ = 4.6 mV/ms, *n* = 0.32. P_50_ Firing in this neuron is 1.0.

Data were acquired with Patchmaster v2.73 software (HEKA Instruments) on a MacPro computer sampling at 20 kHz with 2.9 kHz low pass Bessel filtering. Evoked responses were produced by stimulating the Schaffer collateral pathway with a monopolar 2 MΩ platinum/iridium stimulating microelectrode (FHC, Bowdoin, ME) placed in *stratum radiatum* approximately 350 *μ*m from the CA1 cell soma (Fig. 1A). Holding current for whole‐cell current clamp was adjusted throughout the experiment such that the resting membrane potential was maintained at −70 ± 4 mV.

### Electrophysiological experimental design and data acquisition

Typical experiments were conducted as follows: AP threshold of the postsynaptic neuron was first determined by successive current injections through the patch electrode. The minimum amount of injected current (5 msec step) which generated consistent single APs was used as the rheobase. Then to construct an initial E‐S curve, an input–output series was performed by stimulating the Schaffer collateral pathway at various stimulation current intensities to evoke subthreshold EPSPs and suprathreshold EPSPs generating APs. In general, this input–output series consisted of 6 to 10 stimuli at four different stimulus intensities (200 *μ*sec, typically 5–300 *μ*A) that evoked different probabilities of spiking. Additional stimulus intensities were used that produce spike probabilities of 0% or 100% to fill in the upper and lower bounds of the E‐S curve. The stimulation electrode current intensity was then adjusted to produce baseline EPSPs (every 20 sec) that evoked APs in the postsynaptic cell 30–40% of the time before plasticity induction.

After baseline EPSPs were established, one of two different plasticity‐inducing protocols was applied. The weak induction pairing protocol (WPP) consisted of 100 stimulations to the Schaffer collateral pathway at 0.33 Hz paired with a postsynaptic AP, produced by a 5 msec rheobase current injection and delayed by 8 msec (Fig. 1B, Campanac and Debanne 2008). The stronger induction protocol was based on theta‐burst stimulation (TBS) that employed four sets of pulse trains separated by 10 sec. Each train consisted of 5 bursts of 5 stimulations at 100 Hz. Each burst was elicited at 10 Hz. Each presynaptic stimulation was paired with an AP‐producing 2 msec rheobase current injection delayed by 14 msec (Rosenkranz et al. 2009). When possible, an additional input/output series was collected at least 30 min following the plasticity inducing protocol. Cell input resistance was monitored by hyperpolarizing current pulses (100 msec, 25 pA) applied 500 msec after the presynaptic stimulus. Data were discarded if input resistance changed more than 20%. Because high‐frequency stimulation of Schaffer collateral afferents could potentially induce plasticity, each experiment represents one cell recording per brain slice.

### Data analysis

Data were analyzed using Igor Pro 6.3 software (WaveMetrics, Inc., Lake Oswego, OR). EPSP slopes were determined over the first 2–4 msec of the linear portion of the EPSP rise. EPSP slope versus AP firing probability curves were constructed utilizing the method described previously (Staff and Spruston 2003; Campanac and Debanne 2008; Fink and O'Dell 2009). EPSP slopes were typically sorted into 0.25–1.0 mV/ms bins. AP firing probability per bin was determined by scoring on a binary scale whether or not an AP was elicited from each EPSP. Average AP probability was then calculated from all EPSPs in a bin. P_50_ Firing values, representing a characteristic shift in EPSP‐spike (E‐S) curves, were determined by locating the EPSP slope at which AP firing probability measured before plasticity induction was 0.5. This EPSP slope was then used to determine the firing probability on the postinduction E‐S curve (see Fig. 1D and E).

Smooth curves shown in Figures 1D and E were generated from fits to a modified Boltzmann equation:

(1) $$P_{\mathrm{AP}}=\frac{1}{\left(1+e^{-\frac{\left(\mathrm{ES}-ES_{50}\right)}{n}}\right)}$$

where *P*_*AP*_ is the probability of AP firing, *ES* is the corresponding EPSP slope, *ES*_50_ is the determined EPSP slope for a 50% probability of AP firing, and *n* is the slope indicating the steepness of the relationship. The smooth curve shown in Figure 6C is generated from a logistic equation fit utilizing the following equation:

(2) $$P_{50}\text{Firing}=\frac{1}{1+{\left(\frac{N_{\mathrm{AP}}}{N_{50}}\right)}^{n}}$$

where *N*_*AP*_ is the number of APs elicited from suprathreshold EPSPs during plasticity induction (*see* Fig. 6A) for a neuron with a particular P_50_ Firing value; *N*_50_ represents the number of APs yielded from the fit that produce a P_50_ Firing value of 0.5; and, *n* is the Hill coefficient representing the steepness of the slope relationship.

### Statistics

Statistical significance was assessed with Igor Pro 6.3 software using Student's or single value t‐test. All errors are represented as mean ± S.E.M. Linear regression was also performed on plots of EPSP slope and P_50_ Firing with Pearson's correlational coefficient being calculated.

## Results

### Two types of plasticity can be elicited by a weak plasticity‐inducing pairing protocol

LTP and long‐term depression (LTD) are forms of plasticity found in many regions of the brain, including the hippocampus, and are characterized by modification of synaptic signal strength (typically EPSPs). However, rapid neurotransmission typically requires that EPSPs travel down dendrites, integrate in the cell soma and depolarize the membrane potential to a point where APs are initiated. This signal transmission from EPSP to AP allows for the possibility of long‐term changes in nonsynaptic channel activity to dynamically modulate signal propagation. Such changes would be reflected by E‐S plasticity.

To assay for E‐S plasticity, the relationship between EPSP strength and AP firing probability must be determined. To accomplish this, whole‐cell current clamp recordings were made on CA1 neurons in acute hippocampal slices (Fig. 1A). Initially, the Schaffer collateral pathway was stimulated to evoke suprathreshold EPSPs generating APs approximately 30–40% of the time (Fig. 1B, left). In this way, both synaptic efficacy and AP firing probability could be monitored throughout the experiment (Fig. 1C). After accumulating baseline data, plasticity was induced using a weak pairing protocol (WPP) in which afferent stimulation was paired with an AP‐producing current injection into the postsynaptic neuron (Fig. 1B right).

In control (saline‐treated) rats after WPP, the time course of EPSP potentiation increased slowly and plateaued within 30 min following induction (Figs. 1C and 3A). The degree of LTP was 123.7 ± 12.9%, (*P* = 0.082, paired t‐test in comparison to baseline responses; *n* = 10 cells from eight animals). This very weak degree of LTP and the slow time course of development can probably be attributed to the rather weak stimulation protocol (0.33 Hz, 100 stimuli) utilized for plasticity induction. AP firing probability also increased after WPP induction (Figs. 1C and 3B). In cells producing significant LTP, an increase in postsynaptic AP firing probability is expected with a concomitant increase in EPSP slope.

To determine if there was a change in extrasynaptic plasticity as a result of the WPP stimulation protocol, AP firing probability was analyzed with respect to EPSP slope to form an E‐S curve (Fig. 1D). Curves were generated for data collected prior to, and 30 min after WPP (see Methods). In baseline data before plasticity induction, there was enough variability in EPSP slope to usually generate a full sigmoidal E‐S curve prior to WPP (Fig. 1D). However, after WPP, many CA1 pyramidal cells recorded from saline‐treated animals elicited APs from suprathreshold EPSPs upon every stimulus of the Schaffer collaterals (at the baseline stimulus intensity), thus preventing the generation of the characteristic sigmoidal relationship (Fig. 1E). This lead us to define a new measure for changes in E‐S coupling, termed P_50_ Firing. P_50_ Firing values measure the AP firing probability from data acquired after the plasticity inducing protocol at the EPSP slope of 50% AP firing from data acquired before induction. In most cases where cells remained stable 30 min after WPP, full E‐S curves could also be generated by varying afferent stimulus intensities and collecting sub and suprathreshold EPSPs. E‐S potentiation is indicated by a P_50_ Firing value above 0.5 and represents a left‐shifting E‐S curve, whereas a right‐shifting E‐S curve would have a P_50_ Firing value below 0.5 and indicate E‐S depression.

### Histological pathology in chronically epileptic rats

**Figure 2:**
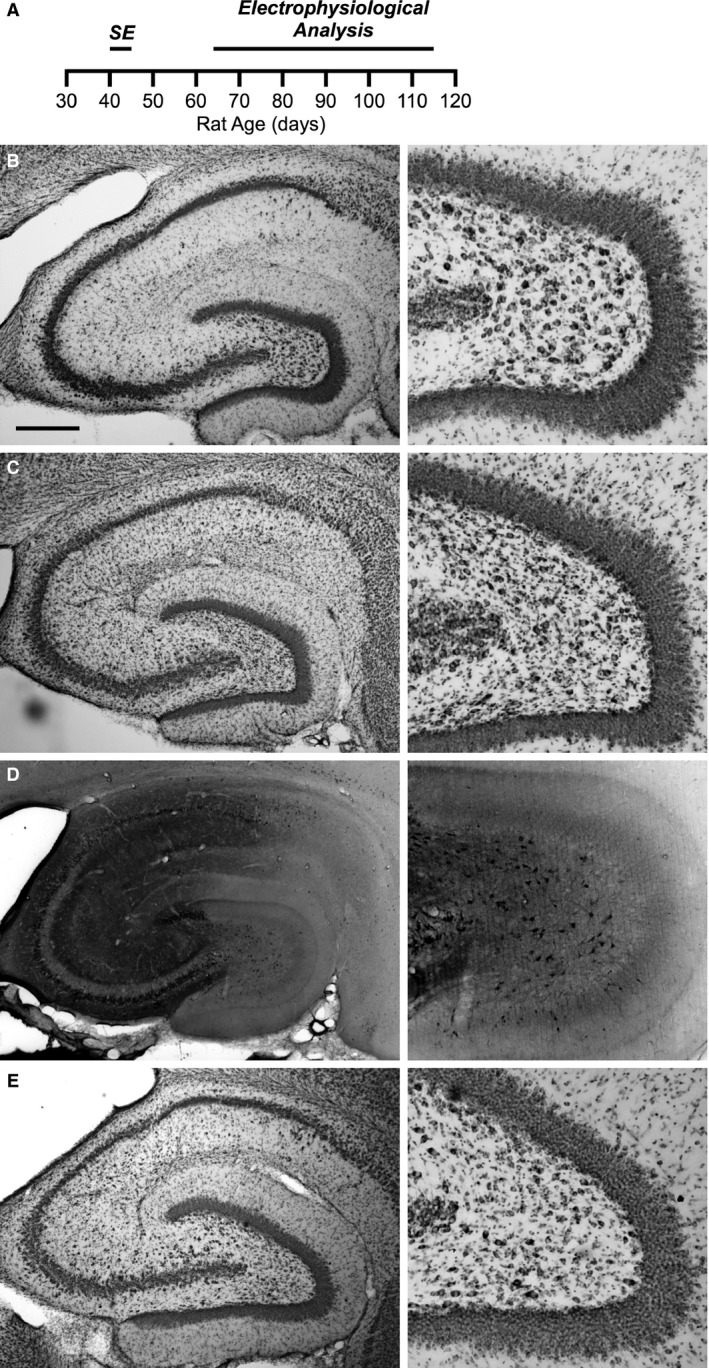
Pilocarpine treatment does not produce extensive neuronal cell death in the CA1 region of the hippocampus. (A) Timeline of the experimental protocol. Pilocarpine or saline is injected in to rats aged P40–P45 producing *status epilepticus* in the pilocarpine‐treated rats. Whole‐cell current clamp recordings from CA1 neurons were done 3–10 weeks later. (B–E) Nissl and Fluoro‐Jade B staining of the hippocampus (*left*) and expanded view of the hilus from the same slice (*right*). (B) Nissl stain 4 days following saline injection shows normal hippocampal structure. (C) Nissl stain 4 days following pilocarpine injection. There is no obvious reduction in *Stratum pyramidale* in CA1 and CA3 regions (*left*). However, characteristic damage resulting from *status epilepticus* can be observed in the hilar region (*right*). (D) Fluoro‐Jade B staining depicted in reverse grayscale of the hippocampus 4 days following pilocarpine injection from the same animal as in (C) Sporadic degenerating neurons (dark neurons) are visible throughout the hippocampus (*left*). Hilar neuron degeneration is also apparent (*right*). (E) Nissl stain 45 days following pilocarpine injection suggests that additional damage to the hippocampus did not occur after the acute response. Lack of degenerating neurons by Fluoro‐Jade B staining confirms this result (data not shown). Scale bar represents 0.5 mm.

To examine the potential impact of the chronic epileptic state on E‐S plasticity we employed the pilocarpine model (Turski et al. 1983a,b; Klitgaard et al. 2002), a rodent model for temporal lobe epilepsy in which chemically induced *status epilepticus* is followed within a few days by spontaneous behavioral seizures (Goffin et al. 2007; Jung et al. 2007; Bumanglag and Sloviter 2008; Curia et al. 2008; Sloviter 2008). The pilocarpine model produces cell death in multiple brain regions (Turski et al. 1983a,b). As we were interested in investigating plasticity changes in a model of chronic epilepsy without the potential confound of extensive cell death, the CA1 region was chosen for this study since less cell death has been reported in this region in comparison to other regions of the hippocampus (Harvey and Sloviter 2005). Nissl and Fluoro‐Jade B staining were performed on 3–4 brains from saline‐ and pilocarpine‐treated rats at 4 days and 45 days following injection to show acute cell death in the hippocampus following *status epilepticus*, and damage occurring during the window of our electrophysiological experiments (Fig. 2B–E). Nissl staining at both time points following saline injection showed relatively normal morphology throughout the hippocampal formation. In addition, there were virtually no Fluoro‐Jade B‐positive cells identified in these animals 45 days following pilocarpine injection (data not shown).

Although there was some variability between animals, Nissl staining at the 4 and 45 day time points after pilocarpine injection did not indicate massive loss of neurons (Fig. 2C and E). Yet, consistent with other reports (Jiao and Nadler 2007; Bumanglag and Sloviter 2008), *status epilepticus* acutely reduced the number of neurons in the hilar region, 4 days after pilocarpine treatment and this was still apparent 45 days after pilocarpine treatment (Fig. 2E). In the CA1 and CA3 regions of the hippocampus, the appearance of degenerating, Fluoro‐Jade B‐positive cells was sporadic and variable between animals (Fig. 2D). Fluoro‐Jade B staining in the hilar region confirmed the presence of degenerating neurons at the 4 day time point (Fig. 2D, *right*). 3 of 4 animals also had a high density of degenerating neurons in the superior blade of the dentate gyrus (Fig. 2D). However, 45 days following pilocarpine injection, virtually no Fluoro‐Jade B positive cells were present (data not shown). The lack of degenerating neurons at the 45‐day time point suggests that severe epileptiform activity and its associated neurodegeneration did not likely occur in the hours prior to hippocampal slice preparation.

### Neurons from pilocarpine‐treated rats on average exhibit reduced E*‐*S potentiation

**Figure 3:**
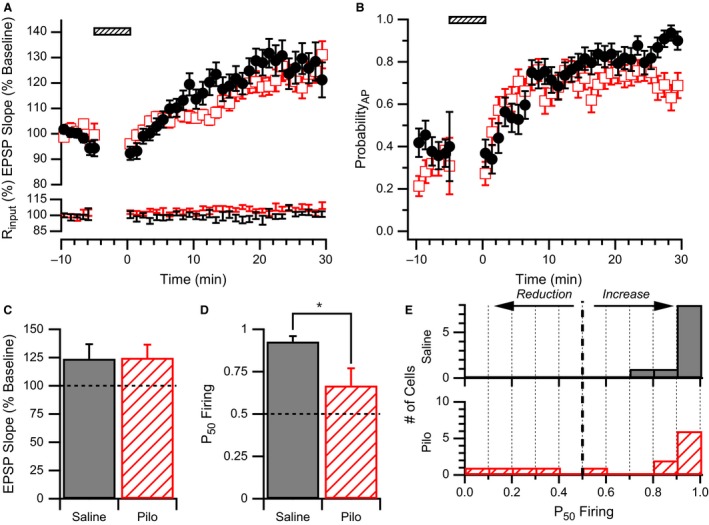
WPP produces strong E‐S potentiation in neurons from saline but not pilocarpine‐treated animals. (A) *Upper Plot*: Average normalized EPSP slope time course for CA1 pyramidal cells from pilocarpine‐treated (open red squares) and saline‐treated (filled black circles) rats. Responses are normalized to the average baseline response before WPP (hatched bar). No difference in average EPSP slope time courses after WPP were observed between groups. *Lower Plot*: Average input resistance normalized to baseline for the same data sets. (B) Average AP firing probability (Probability_AP_) time course for CA1 pyramidal cells from pilocarpine‐treated (open red squares) and saline‐treated (filled black circles) rats. No differences in average AP firing probability time courses were observed. (C) LTP induced by WPP is similar in neurons from saline and pilocarpine (Pilo) treated rats. EPSP slopes measured 30 min after WPP were normalized to their respective baseline values before WPP. (D) E‐S plasticity as measured by P_50_ Firing values in cells from pilocarpine‐treated rats was significantly lower than saline‐treated rats following WPP. * indicates significance *P* < 0.03. (E) E‐S plasticity is highly variable in CA1 cells from pilocarpine‐treated rats. Histogram showing that P_50_ Firing values analyzed from CA1 pyramidal cells from saline‐treated rats (solid) are clustered at high P_50_ Firing values, whereas those from pilocarpine‐treated rats (hatched) are more distributed.

Our electrophysiological studies were conducted 3–10 weeks after saline or pilocarpine injection, ensuring that chronic epilepsy is achieved without acute residual effects from *status epilepticus* (Fig. 2A, timeline). Figure 3A shows the average time course for LTP of recorded CA1 neurons from saline‐ and pilocarpine‐treated rats. Neurons from pilocarpine‐treated rats showed a trend for development of very weak LTP measured 30 min following WPP (paired comparison to baseline: 124.8 ± 11.6%, *n* = 13 cells, *n* = 8 animals, *P* = 0.052). In addition, the degree and time course of LTP development was similar to neurons tested from saline‐treated control animals (unpaired t‐test, *P* = 0.96; Fig. 3A and C). AP firing probability of the postsynaptic cell was also found to rise slowly over a similar time course (Fig. 3B). Although the rats with ongoing epilepsy trended toward lower firing probabilities (saline: 0.78 ± 0.12, *n* = 10 cells from eight animals; pilocarpine 0.69 ± 0.11, *n* = 13 cells from eight animals), they did not differ statistically from controls (Fig. 3B, unpaired t‐test, *P* = 0.6).

However, when measuring E‐S coupling changes that are normalized for changes in EPSP slope before and after WPP (Campanac and Debanne 2008), P_50_ Firing determined from CA1 neurons of saline‐treated rats had values approaching 1 (0.93 ± 0.03, *n* = 10 cells from eight animals), indicating a dramatic increase (left shift) in E‐S coupling (Fig. 3D), despite only a nominal increase in EPSP slope (Fig. 3C). Yet in CA1 neurons from pilocarpine‐treated rats, we found that P_50_ Firing produced by WPP was significantly decreased relative to saline (0.67 ± 0.10, *n* = 13 cells from eight animals, unpaired t‐test *P* < 0.03; Fig. 3D).

The distribution of P_50_ Firing values from individual cells of control animals uniformly underwent large increases in E‐S coupling (P_50_ Firing values approximated 1; Fig. 3E). In contrast, pilocarpine treatment yielded a profile of P_50_ Firing values that were distributed across a large range including a subset of cells that exhibited strong E‐S potentiation similar to control (P_50_ Firing > 0.75). Such variability in P_50_ Firing values could arise from the inherent variability in the pilocarpine model which has been observed with regards to hippocampal circuitry remodeling, neuronal death, and frequency of seizures (Fig. 2; Cavalheiro et al. 1991; Glien et al. 2001; Morimoto et al. 2004). Although the span of time between pilocarpine or saline treatment and the final electrophysiological assay was 3–10 weeks and could be a source of variability, there was no correlation between the age of the rat at the time of assay and P_50_ Firing values for neurons of either group (saline: *r* ^2^ = 0.012, *P* = 0.76, *n* = 10 cells from eight animals; pilocarpine: *r* ^2^ = 0.014, *P* = 0.69, *n* = 13 cells from eight animals).

### E‐S potentiation is distinct from LTP

**Figure 4:**
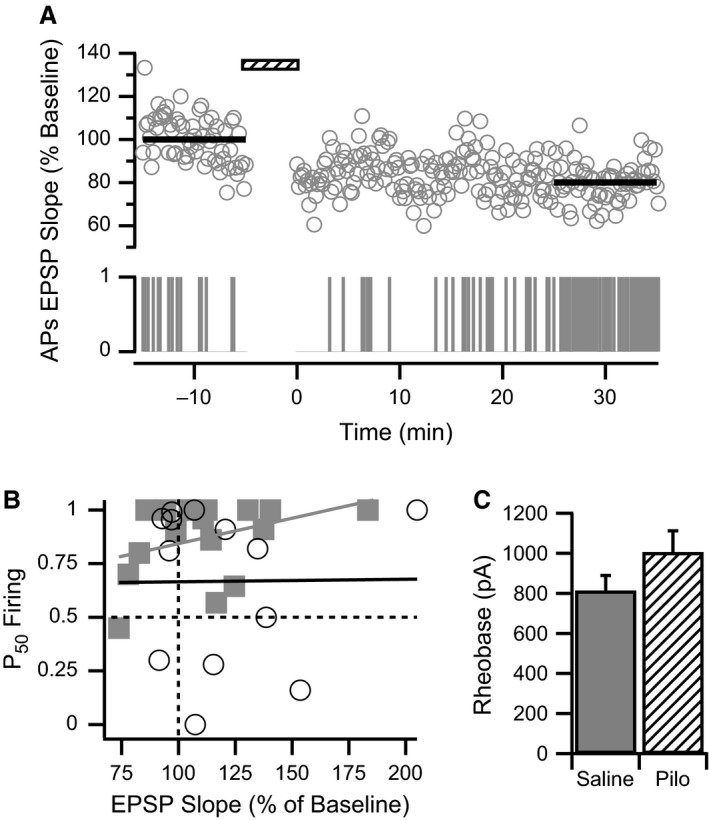
E‐S plasticity is dissociated from synaptic plasticity. (A) E‐S potentiation can be observed concurrently with LTD. Example shows concurrent time courses of normalized EPSP slope (upper graph) and a raster plot of AP firing (lower graph) from a CA1 pyramidal cell from a saline‐treated rat before and after WPP (hatched bar). Averaged EPSP slopes (thick black lines) indicate approximately 20% LTD after WPP. However, the AP Firing raster plot (APs) indicates a gradual increase in AP firing after WPP. (B) Synaptic plasticity and E‐S plasticity are not correlated. P_50_ Firing is plotted against normalized EPSP slopes after WPP for each CA1 neuron from pilocarpine‐treated (open circles) and saline‐treated (filled squares) rats. Vertical dashed line indicates no synaptic plasticity, whereas horizontal dashed line indicates no E‐S plasticity. Correlation for CA1 cells from control (gray line) and pilocarpine‐treated (black line) rats, respectively: *P* = 0.21, *r* ^2^ = 0.095, *n* = 18 from 14 animals; *P* = 0.64, *r* ^2^ = 0.021, *n* = 13 from 8 animals. (C) Rheobase measurement of minimal current injection required to reliably evoke a single AP is similar for CA1 cells from saline‐treated and pilocarpine‐treated (Pilo) animals.

E‐S potentiation is often observed in conjunction with LTP induction (for review: Daoudal and Debanne 2003; Zhang and Linden 2003). The slow time course of development of the left‐shift in E‐S curves in saline‐treated animals (data not shown) reflects the slow time course of LTP development (Fig. 3A). While E‐S potentiation could be coupled to LTP, the robust E‐S potentiation observed for neurons from saline‐treated rats (Fig. 3D) in the absence of significant LTP (Fig. 3C) suggests that LTP and E‐S potentiation may be distinct cellular phenomena. We observed that E‐S potentiation was not always aligned with LTP. For instance, some cells could undergo LTD in response to WPP but still exhibit an increase in AP firing (Fig. 4A). To evaluate the relationship between E‐S plasticity and synaptic plasticity produced by WPP, P_50_ Firing values were plotted against changes in EPSP slope for each cell analyzed from either saline‐treated or pilocarpine‐treated animals (Fig. 4B). Linear regression analysis showed there to be no correlation between EPSP slope and P_50_ Firing values. These findings support the conclusion that E‐S plasticity and synaptic plasticity can be elicited independently.

To evaluate if reduced E‐S potentiation in neurons from chronically epileptic animals (Fig. 3D) could result from a change in active conductances, rheobase current was measured. CA1 pyramidal neurons from pilocarpine‐treated rats tended to require stronger current to evoke single APs in comparison to cells from saline‐treated rats; however, no statistically significant effect on rheobase was observed (saline: 818.2 ± 69.8 *μ*A, *n* = 10 cells from eight animals; pilocarpine: 1007.7 ± 107.1 *μ*A, *n* = 13 cells from eight animals; Fig. 4C). Furthermore, no correlation between P_50_ Firing values and rheobase measurements for individual cells within either experimental group was found (saline: *r* ^2^ = 0.19, *P* = 0.21, *n* = 10 cells from eight animals; pilocarpine: *r* ^2^ = 0.0028, *P* = 0.86, *n* = 13 cells from eight animals). P_50_ Firing values for individual cells were also compared to the input resistance recorded for each cell. Again, no correlation was found for either saline‐ or pilocarpine‐treated groups (saline: *r* ^2^ = 0.0027, *P* = 0.89, *n* = 10 cells from eight animals; pilocarpine: *r* ^2^ = 0.14, *P* = 0.22, *n* = 13 cells from eight animals).

**Figure 5:**
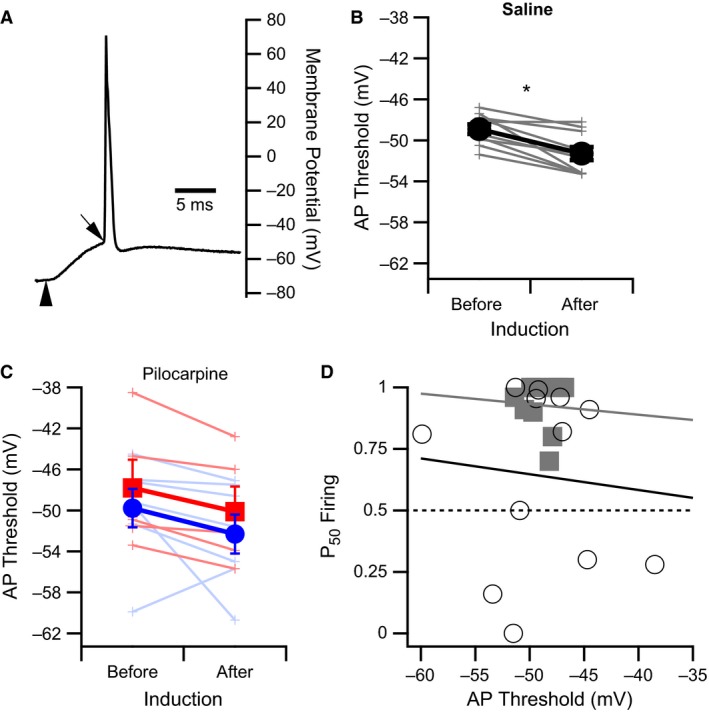
Reduction in AP threshold after WPP is similar for CA1 neurons from saline‐ and pilocarpine‐treated rats. (A) Representative trace of an AP evoked from a suprathreshold EPSP generated from Schaffer collateral stimulation (vertical arrowhead) in a CA1 pyramidal cell from a saline‐treated rat. Diagonal arrow indicates the inflection point defining AP threshold used to generate the data shown in *B* and *C*. (B and C) AP thresholds measured from CA1 pyramidal cells from saline‐treated rats (B) and pilocarpine‐treated rats (C) before and 30 min after WPP. Lines indicate paired measurements. Filled symbols indicate the average values. A slight hyperpolarizing shift in the means for both experimental groups was observed (filled circles). * indicates a significant difference of *P* < 0.001 for data from saline‐treated rats. (C) AP thresholds measured from CA1 pyramidal cells from pilocarpine‐treated rats have been grouped according to P_50_ Firing values. Blue lines and symbols indicate cells in which the P_50_ Firing values > 0.5, whereas red lines and symbols indicate cells with P_50_ Firing values ≤ 0.5. There was no significant difference in AP thresholds before and after WPP for each group. However, when grouped together the p value was less than 0.05. (D) No correlation was found between P_50_ Firing values and AP threshold for CA1 neurons from saline‐treated (filled squares and black line) or pilocarpine‐treated (open circles and gray line) rats. Dashed line indicates no change in E‐S plasticity after WPP.

APs evoked from suprathreshold EPSPs rather than from current injection of somatic origin may more accurately represent physiological conditions that contribute to E‐S plasticity. Therefore, we also determined thresholds for APs elicited from EPSPs by defining the inflection point that differentiates the EPSP from the AP utilizing the first derivative of the trace (Fig. 5A). The variance in the AP threshold was significantly larger in neurons from pilocarpine‐treated rats in comparison to saline‐treated rats (two‐tailed F‐test, *P* < 0.001, *n *= 10 cells from eight saline‐treated animals; 13 cells from eight pilocarpine‐treated animals). Yet, no difference was found in mean AP threshold values of CA1 cells between saline‐treated and pilocarpine‐treated rats. Nevertheless, the WPP induction protocol did result in a slight hyperpolarizing shift in neurons from each group of animals (saline‐treated: before WPP −48.9 ± 0.5 mV, after WPP: −51.3 ± 0.6 mV, *n* = 10 cells from eight animals, paired t‐test *P* < 0.001, Fig. 5B; pilocarpine‐treated: before WPP: −49.0 ± 5.3 mV, after WPP: −51.4 ± 5.1 mV, *n* = 12 cells from eight animals, paired t‐test *P* < 0.05; Fig. 5C, all data).

While the slight hyperpolarizing shift in AP threshold could yield a leftward shift of the E‐S curve, other factors likely dominate (see Discussion). In support of this, no correlation between P_50_ Firing values and baseline AP threshold values was found for individual neurons in either experimental group (saline: *r* ^2^ = 0.0038, *P* = 0.87, *n* = 10 cells from eight animals; pilocarpine: *r* ^2^ = 0.0086, *P* = 0.77, *n* = 12 cells from eight animals; Fig. 5D). Since P_50_ Firing values and AP threshold values for neurons tested from the pilocarpine‐treated rats were highly variable, we determined if low P_50_ Firing values segregated with a particular range of AP thresholds, or WPP‐induced shifts in AP threshold (Fig. 5C). To test this, AP threshold data were grouped based on whether E‐S potentiation was observed (P_50_ Firing > 0.5) or E‐S depression was observed (P_50_ Firing ≤ 0.5). Figure 5C indicates significant overlap of these two groups suggesting that the AP threshold values do not segregate based on E‐S potentiation or depression.

### Stimulation requirements for E*‐*S potentiation

A Hebbian increase in synaptic strength requires almost coincident pre‐ and postsynaptic activity (Shepherd 1990). The WPP has these elements: presynaptic axon stimulation and postsynaptic depolarization by current injection. Yet, both elements may not be required to promote E‐S potentiation, and differences may exist between normal and chronically epileptic animals. To explore the stimulation requirements for E‐S potentiation, CA1 pyramidal cells were subjected to a modified WPP induction protocol where only Schaffer collateral stimulation or postsynaptic somatic current injection was applied. Table 1 shows that Schaffer collateral stimulation alone did not cause an E‐S shift (i.e. P_50_ Firing ~0.5). In contrast, postsynaptic depolarization alone induced E‐S potentiation. In addition, as few as 10 postsynaptic somatic depolarizations that produced APs yielded a robust leftward shift in the E‐S curve (Table 1). We also cataloged the number of cells that exhibited a decrease in E‐S coupling as indicated by a P_50_ Firing value less than 0.5. Approximately one‐third of neurons from pilocarpine‐treated rats that underwent WPP, and one‐third of neurons from either animal group that received only afferent stimulation during induction, exhibited decreased E‐S coupling (Table 1). These findings suggest that afferent input may mediate decreases in E‐S coupling and counteract the robust E‐S potentiation induced by postsynaptic AP firing.

**Table 1:** Stimulation requirements for E‐S potentiation in CA1 Cells from normal or chronically epileptic rats

Treatment	Induction protocol (*WPP*)		P_50_ Firing	P_50_ Firing < 0.5^a^ (*Decreased E‐S Coupling*)
	Schaffer collateral Stimuli (*number*)	Postsynaptic current injections (*number*)		
Pilocarpine	100	100	0.67 ± 0.10	4 (4)/13 (8)
Saline			0.93 ± 0.03	0/10 (8)
Pilocarpine	100	0	0.53 ± 0.13	2 (2)/6 (3)
Saline			0.67 ± 0.17	2 (2)/7 (3)
Pilocarpine	0	100	0.76 ± 0.08	0/4 (3)
Saline			0.72 ± 0.12	1 (1)/8 (4)
Pilocarpine	0	10	0.83 ± 0.08	0/7 (3)
Saline			1.0 ± 0.0	0/3 (2)

a Number of cells – The left value represents cells with decreased E‐S coupling after the induction protocol and the right value represents the total number of cells per group. Number of animals is given in parentheses.

**Figure 6:**
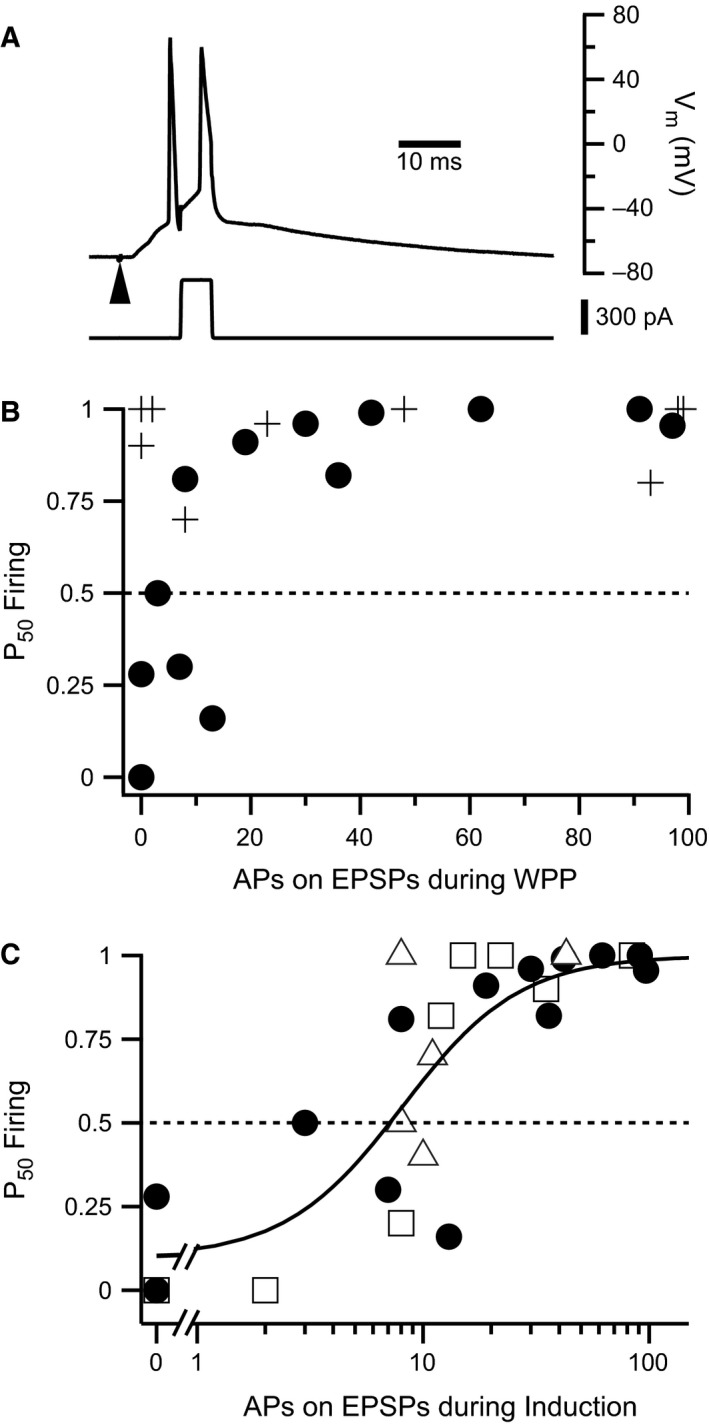
E‐S potentiation is dependent on number of APs elicited during plasticity induction. (A) Example of a trace from the WPP protocol where APs were evoked from both the suprathreshold EPSP and as a result of current injection. (B) Plot shows P_50_ Firing against number of APs evoked by suprathreshold EPSPs during WPP. P_50_ Firing for CA1 neurons from pilocarpine‐treated rats (filled circles) increased with APs evoked by suprathreshold EPSPs, whereas cells from saline‐treated rats did not (crosses). (C) P_50_ Firing exhibits a dose–response relationship with respect to APs evoked from suprathreshold EPSPs. Different symbols reflect neurons subjected to different plasticity induction protocols (*see Table I*). P_50_ Firing values for CA1 neurons from: pilocarpine‐treated rats subjected to WPP (filled circles) or afferent Schaffer collateral stimulation only (open triangles). Open squares represent P_50_ Firing values for CA1 neurons from saline‐treated rats subjected to Schaffer collateral stimulation only. Solid line indicates fit to a logistic equation (Equation 2) for all points. This analysis indicates that greater than 8.1 APs elicited from EPSPs are required to cause E‐S potentiation under these plasticity‐inducing conditions: *N*_50_ = 8.1, *n* = 1.7. Dashed line indicates no change in E‐S coupling after the plasticity inducing protocol.

Because afferent stimulus strength was set to elicit APs 30–40% of the time, during WPP APs were sometimes evoked by suprathreshold EPSPs in addition to APs being triggered by every rheobase current injection (e.g. Fig. 6A). In CA1 pyramidal cells from pilocarpine‐treated animals, P_50_ Firing values increased with an increasing number of APs elicited from suprathreshold EPSPs during WPP. In contrast, CA1 cells from saline‐treated animals yielded P_50_ Firing values near 1 regardless of the number of APs elicited from suprathreshold EPSPs (Fig. 6B). We observed that the combined data from both experimental groups (with the exception of WPP data from saline‐treated rats) could be fit by a Logistic equation. This analysis suggests that 8.1 APs elicited from EPSPs were required to convert the E‐S depressive effect of afferent stimulation into E‐S potentiation (Fig. 6C). These findings corroborate data in Table 1 and demonstrate the relative importance of postsynaptic APs for the induction of E‐S potentiation, and that animals in a chronic epileptic state had reduced sensitivity for E‐S potentiation.

### E*‐*S potentiation requires elevated intracellular calcium

**Figure 7:**
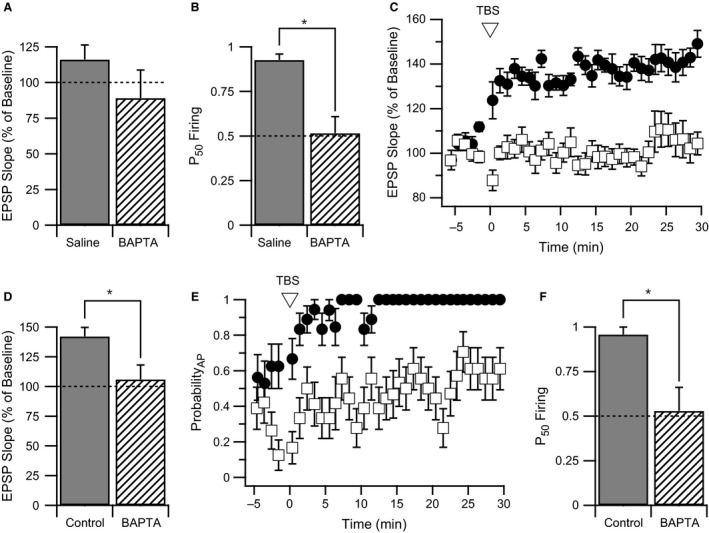
E‐S potentiation and LTP are both dependent on intracellular calcium elevation. A and B. Average normalized EPSP slope change (A) and P_50_ Firing (B) 30 min after WPP for CA1 pyramidal neurons filled with either normal K‐gluconate intracellular recording solution (solid bars) or K‐gluconate solution containing 30 mM BAPTA (hatched bars). Data of saline‐treated controls for change in EPSP slope and P_50_ Firing are the same as Figures 3C and D, respectively. No significant difference in EPSP slope change was observed between groups; however, a significant decrease in P_50_ Firing was observed. * indicates a *P* < 0.01. (C) EPSP slope time course averages for control and BAPTA‐treated cells following theta burst stimulation for LTP induction (TBS). In control cells (filled circles), a robust EPSP slope potentiation was observed. With 30 mM BAPTA intracellular solution (open squares), EPSP slope potentiation was abolished. (D) Bar graph of EPSP slope change at 30 minutes following TBS for control and BAPTA‐treated CA1 cells. * indicates a *P* < 0.05. (E) AP probability time course for control (filled circles) and BAPTA‐treated (open squares) CA1 cells following TBS. In control cells, AP probability rapidly climbs to 100% following TBS. An intracellular solution containing 30 mM BAPTA prevented an increase in AP probability. F. Bar graph of P_50_ Firing in control and BAPTA‐treated CA1 cells following TBS. * indicates a *P* < 0.05.

One major impact of back‐propagating APs is to increase levels of free intracellular calcium (Spruston et al. 1995). To test if E‐S potentiation is a calcium‐dependent process, we conducted a WPP experiment in which the intracellular recording solution contained 30 mmol/L BAPTA, a calcium chelator. The BAPTA‐containing postsynaptic CA1 cells failed to produce LTP (Fig. 7A). Although this result was not significantly different from control cells (123.9 ± 12.9%, *n* = 10 cells from eight animals; BAPTA: 89.2 ± 19.6%, *n* = 5 cells from three animals, *P* = 0.2), a significant difference was not expected since the WPP protocol does not yield strong LTP in control cells (Figs. 3C and 7A). In contrast, the impact of BAPTA on E‐S potentiation was much more pronounced, with a P_50_ Firing value of ~0.5 with low variability, indicating that E‐S coupling was unchanged after WPP (P_50_ Firing: saline = 0.93 ± 0.10, *n* = 10 cells from eight animals; BAPTA = 0.52 ± 0.09, *n* = 5 cells from three animals, *P* < 0.01; Figs. 3D and 7B).

To validate that E‐S potentiation is a calcium‐dependent process, we used a theta burst stimulation (TBS) protocol that produces more robust LTP by delivering a total of 100 pairings of afferent stimulation with postsynaptic current injection in four sets of burst trains. Utilizing this protocol, an average time course for LTP indicated that a 40% potentiated response was achieved in neurons from control rats within 10–15 min of induction (Fig. 7C). A clear block of LTP (*P* < 0.05) was observed in the time course of BAPTA containing neurons and the degree of LTP measured 30 min after TBS was 106.0 ± 12.1% (*n* = 6 cells from six animals) in comparison to 142.1 ± 7.5% for control cells (*n* = 6 cells from six animals; Fig. 7D). AP probability also increased with plasticity induction utilizing TBS (Fig. 7E). In control cells, AP probability stabilized after 12 min and APs were elicited on every EPSP (Probability_AP_ = 1). In contrast, neurons containing intracellular BAPTA had not exceeded an AP probability of 0.6 after 30 min. These results for changes in excitability following TBS were also reflected in a significant decrease in P_50_ Firing values from 0.96 ± 0.04 for control cells (*n* = 6 cells from six animals) to 0.53 ± 0.13 for cells containing BAPTA (*n* = 6 cells from six animals, *P* < 0.05). These experiments show that for both weak and robust plasticity inducting protocols, calcium was required for E‐S potentiation.

## Discussion

### Heterogeneity of E*‐*S potentiation in the chronically epileptic animal

E‐S potentiation was first described with the earliest publications on LTP (Bliss and Lømo 1973) and has been postulated to serve as an additional postsynaptic point for modulation of rapid neuronal signaling throughput (Daoudal and Debanne 2003). Surprisingly, few studies measure E‐S potentiation in single neurons (Staff and Spruston 2003; Campanac and Debanne 2008; Fink and O'Dell 2009). Most measurements of this phenomenon have been made by extracellular recording, where population spike amplitudes were compared with field EPSP slopes before and after a plasticity inducing stimulus protocol (for hippocampal CA1 region: Andersen et al. 1980; Abraham et al. 1987; Fink and O'Dell 2009). Early reports indicated that GABAergic feed‐forward inhibition might play a role in E‐S plasticity (Abraham et al. 1987; Chavez‐Noriega et al. 1989), but later field recording studies indicated that E‐S potentiation was still observed in the presence of GABA_A_ receptor blocker picrotoxin (Hess and Gustafsson 1990; Asztely and Gustafsson 1994). Since field recordings measure responses from populations of neurons, these findings are difficult to interpret from a cellular perspective. An increase in excitability could result from more synchronous firing, more neurons being activated, or a genuine increase in efficacy of firing of individual neurons. In intracellular studies, results showed that a majority of neurons exhibited E‐S potentiation when LTP was observed after tetanic stimulation; however, in cells where LTP was not observed after tetanus, a majority of neurons exhibited E‐S depression (Chavez‐Noriega et al. 1990).

In this study, we found that chronically epileptic, pilocarpine‐treated rats exhibited LTP at Schaffer collateral‐CA1 synapses similar to controls (Figs. 1C, 3A and C), but did not exhibit E‐S potentiation (Fig. 3D). The disruption of E‐S potentiation following pilocarpine treatment resulted from the increased heterogeneity of shifts in E‐S coupling, since approximately half of CA1 cells from pilocarpine‐treated rats had P_50_ Firing values > 0.8 as compared with 100% in saline‐treated rats (Fig. 3E). In two of three instances where multiple neurons were recorded from the same pilocarpine‐treated animal, an increase in E‐S coupling was observed in one neuron and a decrease in E‐S coupling observed in a different neuron. This indicates that heterogeneity in P_50_ Firing values did not arise from variability in the severity of the chronic epileptic state, but rather variability in affected neurons within each epileptic animal. Also in support of this, heterogeneity of P_50_ Firing values did not appear to result from major damage to the hippocampus (Figs. 2C–E), since only sporadic damage in the CA1 and CA3 fields was observed. Additionally, the absence of Fluoro‐Jade B‐positive cells 45 days after pilocarpine injection indicates that there is no evidence of acute degeneration long after the initial insult. This suggests that seizure‐induced cell death is not occurring during the time window of our electrophysiological study.

E‐S potentiation has previously been reported for the CA1 region of hippocampi from both control and chronically epileptic rats (Bernard and Wheal 1996; El‐Hassar et al. 2007). In these studies, E‐S potentiation occurred regardless of whether LTP or LTD was induced. These findings may be consistent with our data, which suggest that LTP and E‐S potentiation are distinct processes (Fig. 4A and B). However, these studies also report an increase in E‐S potentiation for epileptic rats, contradicting our finding of reduced E‐S potentiation in pilocarpine‐treated epileptic rats (Fig. 3D). It should be noted that E‐S relationships determined previously (Bernard and Wheal 1995b, 1996; El‐Hassar et al. 2007) resulted from measurement and analysis of ensembles of neurons (field EPSPs and population spikes) which would not detect the heterogeneity in E‐S plasticity of individual neurons (Fig. 3E).

### Mechanistic differences between LTP and E*‐*S potentiation

Since protocols utilized to induce LTP are also used to induce E‐S potentiation, whether or not E‐S potentiation is mechanistically distinct from LTP needed to be determined. Bliss and Lømo (1973) concluded from the heterogeneity of field EPSPs and population spike responses in the dentate gyrus, that E‐S potentiation might be distinct from LTP. Figure 4B also shows heterogeneity of responses. For example, several neurons from saline‐treated rats expressed E‐S potentiation (P_50_ Firing > 0.75), but no significant LTP. Conversely, two neurons from saline‐treated rats exhibited LTP (EPSP slope > 115%), but showed little E‐S potentiation (P_50_ Firing ~ 0.6) (Fig. 4A and B). Similar results have also been found in CA1 cells utilizing intracellular recording (Chavez‐Noriega et al. 1990). Furthermore, several neurons from chronically epileptic rats exhibited significant LTP, but had E‐S relationships that ranged from highly potentiated (P_50_ Firing > 0.75) to significantly reduced (Fig. 4B). A lack of correlation for the relationship between EPSP slope change and P_50_ Firing for cells from both control and chronically epileptic animals suggests that E‐S potentiation is, at least partially, distinct from modulation of synaptic signaling.

The WPP protocol used to induce E‐S potentiation is virtually identical to the positive pairing protocol utilized by Campanac and Debanne (2008). Our results indicate average LTP of 124% for saline‐treated young adult Sprague–Dawley rats (Fig. 3C), whereas the earlier study on juvenile Wistar rats reports a slightly higher value (129%, Campanac and Debanne 2008). Any difference observed may be attributed to age and/or strain. In addition, both Campanac and Debanne and our study (Fig. 1D) report an average left‐shift in E‐S coupling after WPP. The earlier study shows a correlation between changes in E‐S coupling and changes in EPSP slope induced by WPP. This may suggest significant overlap between the mechanism for producing synaptic plasticity and E‐S plasticity (Campanac and Debanne 2008). This is in apparent contrast with our observations that E‐S potentiation and LTP are at least partially distinct (Fig. 4B). However, changes in E‐S coupling as analyzed by the Campanac and Debanne study are based on measuring mean AP firing probability before and after WPP. AP firing probability is dependent on EPSP slope. An increase in EPSP slope induced by WPP (i.e., LTP) will also increase mean AP firing probability (see also Fig. 3A and B). Thus, these measures of LTP and E‐S potentiation might be biased toward a positive correlation. Our method of assessing E‐S plasticity by generating E‐S curves before and after WPP, removes this bias. While we do see examples of LTP and E‐S potentiation in some cells, our method of normalizing AP firing probability to EPSP slope (Fig. 1D), may be the reason a correlation between E‐S plasticity and LTP was not observed in our study. (Fig. 4B).

Previous work has shown that changes in intrinsic excitability can occur as a result of a plasticity‐inducing protocol like TBS (Aizenman and Linden 2000; Armano et al. 2000; Cudmore and Turrigiano 2004; Frick et al. 2004; Xu et al. 2005; Rosenkranz et al. 2009). These studies differ in age of animals, and measures of intrinsic excitability and the brain regions examined, which may prevent direct comparison with this study. Many studies in the CA1 region of the hippocampus utilize TBS to induce plasticity in conjunction with somatic current injections to evaluate changes in excitability (Frick et al. 2004; Xu et al. 2005; Rosenkranz et al. 2009). To show that a mechanism related to changing intrinsic excitability is distinct from mechanisms related to LTP, the sensitivity of inducing E‐S potentiation can be tested by employing a weak induction protocol where little or no LTP is elicited. Another difference between these two mechanisms can be identified from differing presynaptic and postsynaptic requirements for induction of the two processes. Such approaches allow for separation of multiple pathways for LTP and other plastic phenomena that potentially overlap with E‐S plasticity.

Utilizing WPP, we were able to detect differences between E‐S potentiation and LTP. The mean degree of WPP‐induced LTP was only 123.9% for cells tested from saline‐treated animals (Fig. 3C). This was not significantly different from 100% (*P* = 0.10, *n* = 10 cells from 8 animals; *single sample t‐test*), indicating very weak synaptic potentiation. Yet, corresponding P_50_ Firing values were highly significant compared to 0.5 (*P* < 0.001, *n* = 13 cells from eight animals; *single sample t‐test*). This indicates that significantly less stimulation is required to produce a leftward (potentiating) shift in E‐S coupling than is required to produce significant LTP. In addition, Figure 6 shows that this leftward shift in E‐S coupling is dependent on the number of action potentials evoked during WPP for neurons from pilocarpine‐treated animals. Although no such relationship is observed for saline‐treated animals (Fig. 6B), we note that APs are elicited in the WPP by either Schaffer collateral stimulation producing suprathreshold EPSPs or by postsynaptic somatic current injection. Thus, induction of E‐S potentiation in neurons from saline‐treated animals may require a lower number of postsynaptic APs than for neurons from chronically epileptic animals induced by pilocarpine treatment. Fink and O'Dell (2009) also observed that as few as 15 APs coupled with postsynaptic depolarization can produce an increase in E‐S coupling in CA1 hippocampal neurons.

The pre‐ and postsynaptic requirements for LTP and E‐S potentiation are also found to be different. LTP in CA1 pyramidal neurons can be induced solely by afferent glutamatergic input delivered at high frequency (e.g., 100 Hz tetanus; reviewed in Bliss and Collingridge 1993), or by paired afferent input with postsynaptic current injection (Gustafsson et al. 1987; Magee and Johnston 1997; Wittenberg and Wang 2006). Either of these induction methods can serve to activate AMPA receptors, depolarize the cell, and thereby allow for the activation of NMDA receptors as well as voltage‐gated calcium channels (for review Malenka and Nicoll 1999; Malenka and Bear 2004). However, Table 1 indicates that the sole requirement for significant E‐S potentiation is postsynaptic somatic current injection that elicits APs.

Theoretically, E‐S potentiation could be caused by a reduction in AP threshold that was initiated by a plasticity‐inducing protocol. WPP produced a significant decrease in AP threshold for CA1 neurons from both saline‐ and pilocarpine‐treated rats (Fig. 5B and C). However, the average AP threshold values before and after plasticity induction for saline and pilocarpine‐treated animals were similar; yet, CA1 pyramidal neurons from saline‐treated rats exhibited significant E‐S potentiation, whereas many cells from pilocarpine‐treated rats did not (Fig. 3D and E). This suggests that the slight decrease in AP threshold does not significantly contribute to E‐S potentiation. In addition, when TBS was used for induction of plasticity, no significant change in threshold for cells from control animals was noted (before induction: −50.4 ± 0.9 mV, after induction −50.9 ± 0.9 mV, *n* = 6 cells from six animals, *P* = 0.31). Also, in juvenile rats, no change in AP threshold was found (Campanac and Debanne 2008). Together, these data suggest that AP threshold may be only partially responsible for shifts in E‐S coupling.

Our finding that LTP and E‐S potentiation with either WPP or TBS require intracellular calcium supports the idea that these two phenomena share some commonality (Fig. 7A–F). E‐S potentiation can be elicited without afferent glutamatergic input (Table 1). This may suggest that an increase in intracellular calcium needed for E‐S potentiation may not be NMDA receptor dependent. Support for this may come from induction of other types of plasticity. Some forms of LTP are not strictly dependent on NMDA receptor activation, and voltage‐gated calcium channels can provide the required calcium influx (Grover and Teyler 1990; Teyler et al. 1994; Cavuş and Teyler 1996; Grover 1998; Kanterewicz et al. 2000). Indeed, in a previous field potential study, E‐S potentiation was NMDA receptor independent (Bernard and Wheal 1995a). In contrast, others have reported that LTP and E‐S potentiation in CA1 pyramidal cells are both NMDA receptor dependent (Campanac and Debanne 2008; Fink and O'Dell 2009). Augmentation of EPSP responses may also be dependent on calcium release from intracellular stores (Harvey and Collingridge 1992; Bortolotto and Collingridge 1993; Mellentin et al. 2007). Such conflicting evidence may indicate the presence of multiple sources for elevating intracellular calcium to induce synaptic plasticity and E‐S potentiation individually or concurrently.

### Significance to epilepsy

Our primary finding is that CA1 pyramidal cells from epileptic pilocarpine‐treated rats exhibited a diverse response, which on average shows reduced E‐S potentiation. This may appear to contradict the notion that chronically epileptic tissue contains circuitry that promotes overexcitation. However, mechanisms that contribute to an imbalance of excitation and inhibition need not be the same as mechanisms that promote learning and memory. For example, overexcitation might generate hippocampal seizures due to hilar neuron loss and synaptic reorganization (Tauck and Nadler 1985; Sloviter 1987; Harvey and Sloviter 2005), whereas memory formation and cognitive deficits may be linked to synaptic plasticity (Bliss and Collingridge 1993). Cognitive deficits have long been associated with epilepsy (Hesse and Teyler 1976; Hu et al. 2005); yet in animal models of epilepsy, LTP is apparent. However, some investigations report a reduction in LTP (Chang et al. 2005; Zhang et al. 2010; Zhou et al. 2011; Kryukov et al. 2016), whereas others report facilitation of LTP (Notenboom et al. 2010; Muller et al. 2013). When the possibility that synaptic and E‐S plasticity vary independently is considered (Fig. 4), the relationship between altered neurophysiology and cognitive deficits in epilepsy becomes more nuanced than the simple disruption of LTP. Thus, it is possible that E‐S potentiation may play a role in the comorbid cognitive deficits in epilepsy.

The precise function of E‐S plasticity in cognition remains uncertain. Although speculative, our data suggest how E‐S plasticity contributes to network function. Enhancement of E‐S coupling increases excitatory drive in the CA1 region; however, high‐frequency stimulation of *stratum radiatum* also can enhance E‐S coupling in parvalbumin‐positive basket cells which also enhances feed‐forward inhibition in the CA1 region (Campanac et al. 2013). Increases in E‐S coupling may modulate the frequency of firing such that action potential firing is enhanced in the 25–50 Hz (gamma) range (Campanac et al. 2013). Theta and gamma oscillation coupling has been associated with learning and memory in the hippocampal formation (Montgomery and Buzsaki 2007; Colgin 2015; Zheng et al. 2016). We have shown that E‐S potentiation was robust in all ten CA1 neurons tested from saline‐treated rats, whereas E‐S potentiation varied widely from P_50_ Firing values of 0 to 1 for animals with ongoing epilepsy. This suggests that cognitive deficits could evolve from the lack of a unified modulatory response (in this case a unified E‐S shift), and thereby prevent CA1 output to the cortex from being synchronized. In computational models, such synchronicity has been associated with reliable signal propagation in feed‐forward networks (Diesmann et al. 1999; Reyes 2003; Jahnke et al. 2013; Han et al. 2015). Although our data, being focused on single cell recording, does not address neuronal synchrony directly, a coordinated increase in CA1 spike probability might produce such an effect. Future work will aid in determining how E‐S potentiation may be functionally related to network signal propagation and cognitive deficits in neurological disease

## **Conflict of Interest**

The authors have no competing interests.
